# Effects of Huanglian-Jie-Du-Tang and Its Modified Formula on the Modulation of Amyloid-β Precursor Protein Processing in Alzheimer's Disease Models

**DOI:** 10.1371/journal.pone.0092954

**Published:** 2014-03-26

**Authors:** Siva Sundara Kumar Durairajan, Ying-Yu Huang, Pui-Yee Yuen, Lei-Lei Chen, Ka-Yan Kwok, Liang-Feng Liu, Ju-Xian Song, Quan-Bin Han, Lei Xue, Sookja K. Chung, Jian-Dong Huang, Larry Baum, Sanjib Senapati, Min Li

**Affiliations:** 1 Neuroscience Research Laboratory, School of Chinese Medicine, Hong Kong Baptist University, Kowloon Tong, Hong Kong; 2 Natural Products Chemistry & Analysis Laboratory, School of Chinese Medicine, Hong Kong Baptist University, Kowloon Tong, Hong Kong; 3 Shanghai Key Laboratory of Signaling and Disease Research, School of Life Science and Technology, Tongji University, Shanghai, China; 4 Department of Anatomy, Li Ka Shing Faculty of Medicine, The University of Hong Kong, Pokfulam, Hong Kong; 5 Department of Biochemistry, Li Ka Shing Faculty of Medicine, The University of Hong Kong, Pokfulam, Hong Kong; 6 School of Pharmacy, The Chinese University of Hong Kong, Shatin, Hong Kong; 7 Department of Biotechnology, Indian Institute of Technology Madras, Chennai, India; Oregon Health and Science University, United States of America

## Abstract

Huanglian-Jie-Du-Tang (HLJDT) is a famous traditional Chinese herbal formula that has been widely used clinically to treat cerebral ischemia. Recently, we found that berberine, a major alkaloid compound in HLJDT, reduced amyloid-β (Aβ) accumulation in an Alzheimer’s disease (AD) mouse model. In this study, we compared the effects of HLJDT, four single component herbs of HLJDT (Rhizoma coptidis (RC), Radix scutellariae (RS), Cortex phellodendri (CP) and Fructus gardenia (FG)) and the modified formula of HLJDT (HLJDT-M, which is free of RS) on the regulatory processing of amyloid-β precursor protein (APP) in an *in vitro* model of AD. Here we show that treatment with HLJDT-M and its components RC, CP, and the main compound berberine on N2a mouse neuroblastoma cells stably expressing human APP with the Swedish mutation (N2a-SwedAPP) significantly decreased the levels of full-length APP, phosphorylated APP at threonine 668, C-terminal fragments of APP, soluble APP (sAPP)-α and sAPPβ-Swedish and reduced the generation of Aβ peptide in the cell lysates of N2a-SwedAPP. HLJDT-M showed more significant APP- and Aβ- reducing effects than berberine, RC or CP treatment alone. In contrast, HLJDT, its component RS and the main active compound of RS, baicalein, strongly increased the levels of all the metabolic products of APP in the cell lysates. The extract from FG, however, did not influence APP modulation. Interestingly, regular treatment of TgCRND8 APP transgenic mice with baicalein exacerbated the amyloid plaque burden, APP metabolism and Aβ production. Taken together, these data provide convincing evidence that HLJDT and baicalein treatment can increase the amyloidogenic metabolism of APP which is at least partly responsible for the baicalein-mediated Aβ plaque increase in the brains of TgCRND8 mice. On the other hand, HLJDT-M significantly decreased all the APP metabolic products including Aβ. Further study of HLJDT-M for therapeutic use in treating AD is warranted.

## Introduction

Extracellular senile plaques and phosphorylated tau-associated intraneuronal neurofibrillary tangles (NFTs) are the two classical microscopic pathologies of Alzheimer’s disease (AD) [1]. Senile plaques comprise a dense core of amyloid-β (Aβ) that is surrounded by dystrophic neurites [1]. Aβ is a 39–43 amino acid proteolytic product of a much larger amyloid precursor protein (APP). APP is an integral membrane protein processed by the proteases α-secretase or β-secretase to produce α-C terminal fragment (CTF-α) or β-C terminal fragment (CTF-β), respectively. These fragments are subsequently cleaved by γ-secretase to produce P3 or Aβ respectively, and a cytoplasmic tail dubbed APP-intracellular domain (AICD) [1]. APP proteolysis also releases soluble forms of APP (sAPPα and sAPPβ), and these soluble APPs may also now be considered biomarkers for AD [2]. On the other hand, monomeric Aβ (4.3 kDa molecular weight) self-assembles into oligomers. These oligomers eventually deposit as large fibrils in extracellular space, which assemble as amyloid plaques [1], [3]. Although the precise mechanisms by which Aβ may induce neurotoxicity are still unknown, several are proposed, including calcium influx, generation of reactive oxygen species (ROS), nitric oxide (NO) production and increased phosphorylation of tau [4].

The US Food and Drug Administration has approved five drugs (i.e., tacrine, donepezil, rivastigmine, galantamine and memantine) for the treatment of AD, but they produce only mild, symptomatic relief and do not halt progression of dementia [5]. Therefore there is a need for alternative drugs for the treatment of AD. One potential source of phytotherapeutic agents is Huang-Lian-Jie-Du-Tang (HLJDT), a traditional Chinese medicine (TCM) achieving popularity for its therapeutic application.

Huang-Lian-Jie-Du-Tang (HLJDT) is a famous TCM formula widely used in treating stroke and dementia. It is composed of four herbs, namely: Rhizoma coptidis (RC) (*Coptis chinensis* Franch, or Huang Lian in Chinese), Radix scutellariae (RS) (*Scutellaria baicalensis* Georgi, or Huang Qin in Chinese), Cortex phellodendri (CP) (*Phellodendron amurense,* or Huang Bai in Chinese) and Fructus gardeniae (FG) (*Gardenia jasminoides* Ellis, or Zhi Zi in Chinese), in a 3∶2:2∶3 dry weight ratio. As stated in the traditional Chinese medicinal book Wai-Tai-Mi-Yao, RC, RS, and CP are major ingredients of HLJDT, and FG functions as an adjuvant constituent to support the effect of the principal ingredients.

HLJDT has been used to treat senile dementia, inflammation, digestive system upsets, and cerebrovascular disease in China [6]. HLJDT has been used to treat various clinical symptoms linked with stroke [7] and with vascular dementia [8] in Japan. In a Japanese clinical study, the addition of HLJDT to yokukan-san (Japanese traditional herbal medicine) exerted the same efficacy as aripiprazole (antipsychotics) in controlling aggressiveness in an Alzheimer’s type dementia patient without any adverse effects [9].

Preclinical reports provide evidence that HLJDT can improve cerebral blood flow; it potently inhibits lipid peroxidation in the brain and thus preserves energy metabolism in the brain [10],[11],[12]. Both ethanolic extracts and aqueous extracts of HLJDT can ameliorate the cognitive impairments induced by cerebral ischemia and central cholinergic dysfunction in animal models [13],[14]. We have recently shown that berberine, a compound in HLJDT, can significantly reduce the Aβ load in a transgenic Alzheimer’s disease model by regulating APP processing [15]. However, the exact mechanism underlying HLJDT-mediated cognitive improvements is not known. In the context of AD, there is a study of HLJDT in AD mice [16]. Qiu et al. [16] reported that HLJDT reduced Aβ plaques and improved memory in APP/PS-1 mice, but the authors did not mention the quantification of Aβ load. Qiu et al. [16] also reported that HLJDT reduced APP mRNA level but did not measure the effect of HLJDT on the protein level of full length (Fl)-APP, Aβ and soluble forms of APP, namely, sAPPα and sAPPβ. In contrast with Qiu et al. [16], we found that HLJDT increased Fl-APP, sAPPα, sAPPβ and intracellular Aβ in N2a mouse neuroblastoma cells expressing APP with the Swedish mutation (N2a-SwedAPP cells). We also found that RS enhances Aβ generation by increasing the protein level of APP. To further prove this we compared the effect of HLJDT with and without RS (modified HLJDT, or HLJDT-M) on APP processing and Aβ load in N2a-SwedAPP cells. We meticulously evaluated the effect of each individual herb of HJLDT on the levels of APP, sAPPα and sAPPβ in order to determine which herb is responsible for increasing APP and Aβ.

## Materials and Methods

### Chemicals and Reagents

Dulbecco's modified Eagle's medium (DMEM), fetal bovine serum (FBS), penicillin, streptomycin and G418 for cell culture were bought from Invitrogen (Carlsbad, CA, USA). Polyvinylidene Fluoride (PVDF) membrane was obtained from Hybond-P, GE Healthcare BioSciences (Piscataway, NJ, USA). Enhanced chemiluminescence (ECL) reagent was purchased from Thermo Scientific (Rockford, IL, USA). Tetramethylbenzidine (TMB) was purchased from BD Biosciences (Sparks, MD, USA), while analytical grade reagents (including ethanol and methanol) were from Sigma–Aldrich (St. Louis, MO, USA) unless otherwise indicated. Berberine, baicalin and baicalein were purchased from Sigma-Aldrich. Palmatine and geniposide were purchased from Aktin Chemicals (Chengdu, China).

Monoclonal β-actin antibody was purchased from Santa Cruz Biotechnology (Santa Cruz, CA, USA). Rabbit polyclonal CT15 antibody against the C-terminus of APP was a gift from Prof. Edward Koo (University of California, San Diego, La Jolla, CA, USA). Amino-terminus anti-Aβ1–17 monoclonal antibody (6E10) and a biotinylated Aβ17–24 (4G8) monoclonal antibody wereordered from Covance (Princeton, NJ, USA). Human anti-sAPPβ Swedish (sAPPβ-sw) monoclonal antibody (clone 6A1) was provided by IBL (Japan). Anti-pAPPThr668 polyclonal antibody was from Cell Signaling (Danvers, MA, USA). Carboxy-terminus biotinylated anti-amyloid bG4(1–40)-5C3 (specific to a peptide corresponding to Aβ40) and bA4(1–42)-8G7 (specific to a peptide corresponding to Aβ42) antibodies were purchased from Nanotools (Teningen, Germany). The streptavidin-conjugated horseradish peroxidase (HRP) was purchased from DAKO (Carpinteria, CA, USA). Aβ40 and Aβ42 peptides were provided by Invitrogen (Carlsbad, CA, USA) and California Peptide (Napa, CA, USA), respectively.

### Plant Extraction

RC, RS, CP and FG were purchased from the Hong Kong Baptist University Mr. & Mrs. Chan Hon Yin Chinese Medicine Specialty Clinic and Good Clinical Practice Centre and from a local Chinese medicine pharmacy. All herbs were identified and authenticated by Prof. Zhong-Zhen Zhao from the School of Chinese Medicine, Hong Kong Baptist University, Hong Kong. Dry materials of the plants were ground into powder. Approximately 10 g of powder of each herb, and of the HLJDT mixture with and without RS, were soaked in 100 mL of 80% ethanol for 1 h, with sonication at a frequency of 120 kHz (SANHO Ultrasonic Engineering Ltd., Hong Kong); then extracted solutions were filtered. This procedure was repeated three times. Thus we employed this solvent to standardize the extraction and to focus on screening. Approximately 300 mL of each extracted solution was collected and, then concentrated by rotary evaporation (EYELA, Tokyo Rikakikai Co., Ltd., Japan) under vacuum in a 60°C water bath. All the extracts were finally subjected to lyophilization (LABCONCO, Laboratory Construction Company, MO, USA) at −40°C under vacuum of 105 μbar. Each yield of plants was powdered and mixed until uniform, and then stored at 4°C for later use. Dimethyl sulfoxide (DMSO) was used as the solvent to dissolve the extract, and it was loaded as the vehicle control for all cell cultures. Although the aqueous extract of HLJDT have been used in some studies, we found that the ethanol extract was ideal to isolate most of the bioactive compounds with higher quantity from HLJDT when compared to aqueous extract (data not shown). It was reported that ethanol can extract higher concentrations of flavonoid, polyphenols and more alkaloid compounds as compared to aqueous extract [17],[18]. Thus we employed this solvent to standardize the extraction and to focus on screening.

### Chromatographic Conditions

**Figure 1:**
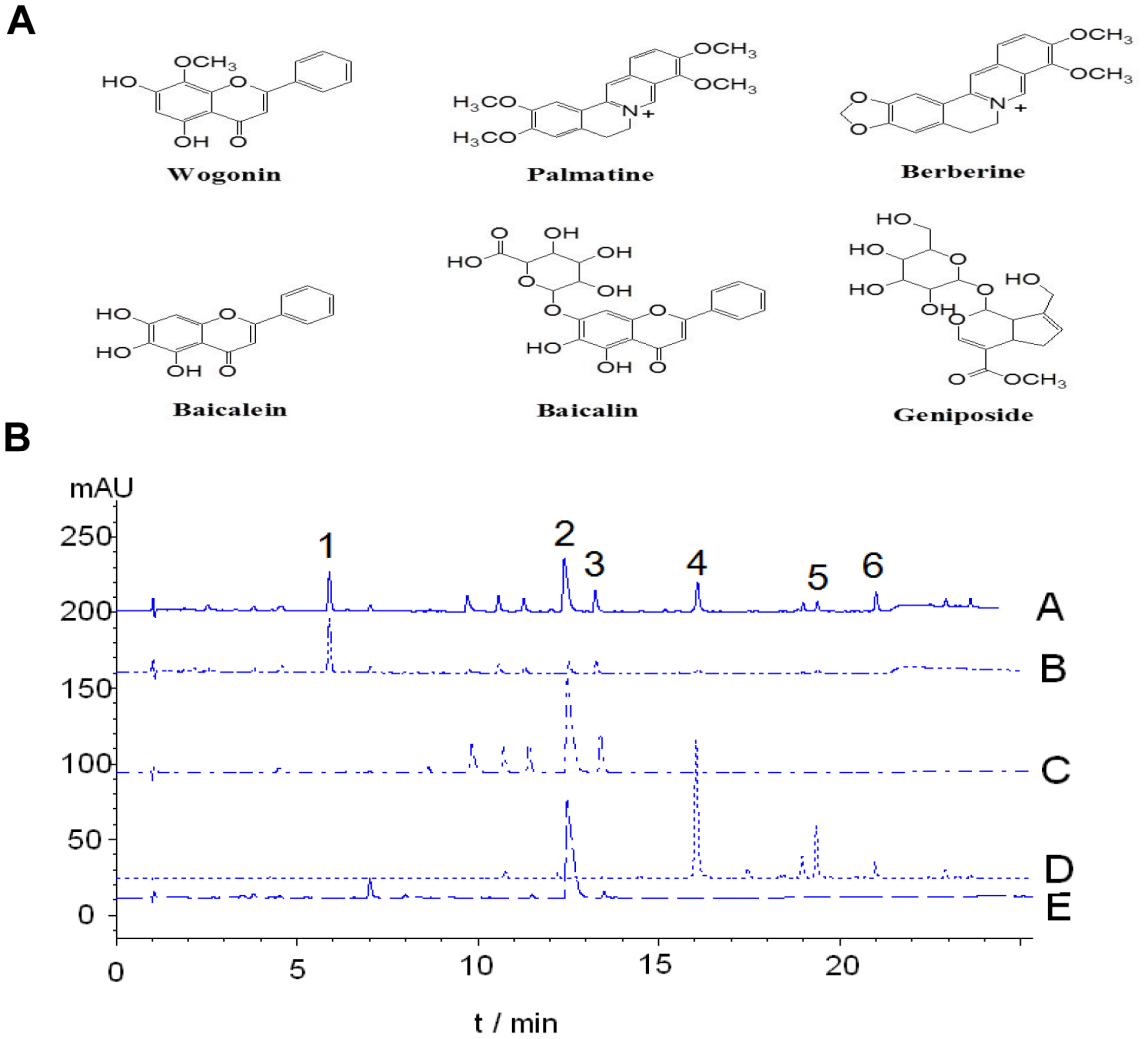
Selected components of Huang-Liang-Jie-Du-Tang (HLJDT). A. Chemical structures of main compounds of Huang-Liang-Jie-Du-Tang (HLJDT). B. Quality analysis of HLJDT (at 245 nm) (A); Fructus gardeniae (FG) (at 245 nm) (B); Rhizoma coptidis (FG) (at 275 nm) (C); Radix scutellariae (RS) (at 275 nm) (D); Cortex phellodendri (CP) (at 275 nm) (E). Peaks of these compounds are shown: 1. Geniposide; 2. Berberine; 3. Palmatine; 4. Baicalein; 5. Baicalin; 6. Wogonin.

HPLC was carried out on an Agilent 1100 series with a G1315A diode array detector (California, USA). An Alltima™ C18 column (250×4.60 mm, particle size 5 μm) was used for separations. HPLC conditions were as follows: eluent A, 0.1% formic acid in H_2_O; eluent B, methanol with a linear gradient elution (0 min, 15% B; 0∼15 min, 15%→38% B; 15∼30 min, 38%→90% B; 30∼30.1 min, 90%→100% B; 30.1∼33 min, 100% B; 33∼33.1 min, 100%→15% B; 33.1∼36 min, 15% B) at a flow of 1 mL/min. Peaks were assigned by matching their retention times with that of each reference compound eluted in parallel with the same mobile phase. The concentrations of the analytes were determined from representative calibration curves. From the HPLC profiles of ethanolic extract of HLJDT and its individual herbs, we have found that, in comparing the concentrations of six known components (geniposide, berberine, palmatine, baicalein, baicalin and wogonin) that can be found in ethanolic extract, berberine is most abundant (6.02%), followed by geniposide (4.01%), baicalin (2.67%), baicalein (1.31%), palmatine (0.867%) and wogonin (0.615%) (Figure 1B; Table S1). Based on the four herbal components of HLJDT, we know that geniposide is contributed by FG; berberine and palmatine are mainly found in RC and CP, respectively; and baicalin, baicalein and wogonin are contributed by RS. Recent studies have shown that berberine, baicalein and geniposide have neuroprotective effects in Alzheimer’s disease models [15],[19],[20]. Therefore, these three compounds (Figure 1A) were used for quantitative analysis of ethanolic extract of HLJDT.

Qualitative identification of 12 active compounds of HLJDT was also performed by ultra-high performance liquid chromatography with quadrupole time-of-flight mass spectrometry (UHPLC-Q-TOF-MS) analysis with positive mode ESI-MS (supplementary method). A database was prepared for the identification of phytoconstituents of HLJDT based upon their molecular masses, calculated m/z values and retention times (Rt) observed in the analysis (Table S1). Representative LC-MS base peak chromatograms (BPC) and extracted ion chromatograms (EIC) are shown in Figure S1. By matching the Rt and m/z values between samples and reference standard solutions (Table S2), eight characteristic peaks of HLJDT were identified in HLJDT sample solutions as geniposide, coptisine, jatrorrhizine, baicalin, palmatine, berberine, baicalein, wogonin, phellodendrine, columbamine, epiberberine and wogonoside were identified based on published articles related to the profiling of chemical components of HLJDT [21],[22].

### Cell Culture

N2a-SwedAPP cells [23] were obtained from Dr. Gopal Thinakaran (University of Chicago, Chicago, IL, USA). N2a-SwedAPP cells were cultured in Dulbecco's modified Eagle's medium (DMEM) and OPTI-MEM medium in a 1∶1 ratio with 5% FBS, 50 μg/mL penicillin, 50 μg/mL streptomycin and 200 μg/mL G418 as previously described [23]. N2a-SwedAPP cells were incubated at 37°C in a 5% CO_2_/95% humidity incubator. Cells were seeded 24 h prior to the treatments. When growth reached close to 90% of confluence, cells were transferred into DMEM with 1% FBS for loading extracts. The final concentration of DMSO in all experiments was 0.1%, and this concentration caused no cytotoxicity.

### Viability Assay

For the viability assay, N2a-SwedAPP cells were seeded in a 96-well plate (7000 cells/well and 5000 cells/well) for 48 h. The exhausted medium was replaced after 24 h with 200 μL DMEM with 1% FBS and various concentrations (0, 3.125, 6.25, 12.5, 25 or 50 μg/mL) of each extract or various concentrations (0, 3.125, 6.25, 12.5 or 25 μM) of berberine, baicalein or geniposide, and cells were incubated at 37°C in 5% CO_2_ for 48 h. Then the media were removed and 100 μL of phenol red-free DMEM containing 3-(4,5-dimethylthiazol-2-yl)-2,5-diphenyltetrazolium bromide (MTT) was added to each well to a final concentration of 0.5 mg/mL and further incubated for 4 h. The MTT-containing medium was removed, and the cell crystals were dissolved using 100 μL of 20% sodium dodecyl sulfate (SDS) in 50% *N*, *N*-dimethylformamide. Finally, optical intensity was measured using a BioRad plate reader at 570 nm and a reference of 620 nm. Experiments were conducted independently three times.

### Detection of Intracellular APP and Soluble APPs in N2a-SwedAPP Cells

To detect the levels of APP metabolic products, the cell lysates and the conditioned media were prepared as described previously by Durairajan et al. [24], with minor modification. N2a-SwedAPP cells were seeded at a density of 4×10^5^/mL in DMEM medium in a 6-well plate and cultured for 1 day. After transfer, cells were treated with different doses of RC, CP, FG and RS for 48 h, then the extracellular media were collected for detection of sAPPα and sAPPβ-sw, and cells were harvested to quantify Fl-APP and β-actin by Western blot analysis, as described previously [24]. For intracellular APP, APP-CTFs and Aβ preparation, cells were washed with ice-cold PBS twice and solubilized in ice-cold RIPA buffer with protease inhibitor cocktail added. Lysates were collected and centrifuged at 16,000×g for 15 min at 4°C. The resulting supernatants were collected, and their protein concentrations were determined with bicinchoninic acid (BCA) protein assay reagent (Thermo Scientific, IL, USA). For sAPPα and sAPPβ-sw detection, the conditioned media were collected and frozen in liquid nitrogen, subjected to lyophilization at −40°C under vacuum of 105 μbar for 2 days in order to remove all liquid. The conditioned media were standardized to total cell lysate protein.

### Animals and Baicalein Treatment

All animal handling experiments were approved by the Hong Kong Baptist University Committee on the Use of Human and Animal Subjects in Teaching and Research (HASC, approval #: 12–13/0030). TgCRND8 mice expressing human APP with Swedish (K670N/M671L) and Indiana (V717F) mutations were used to evaluate the effect of baicalein on Aβ. Heterozygous TgCRND8 mice on a C57BL/6J background [25] were used to breed a colony of experimental animals. The TgCRND8 mouse is an early onset model of AD with visible Aβ deposits in the brains of the animals starting from 3 months of age [25]. The animals were housed in a controlled environment under a 12/12 h light/dark cycle. They were allowed free access to food and water. The oral administration of baicalein (25 mg/kg/d) was commenced at 2 months of age and ended at 5 months before being killed. We tested a single dose (25 mg/kg/d) of baicalein in our animal study because in our pilot study we did not find any difference in the brain levels of baicalein between doses of 25 and 50 mg/kg/d (data not shown). Control mice were orally gavaged with tap water only for the same administration period. At the conclusion of the treatment period, mice were deeply anesthetized by chloral hydrate and perfused with phosphate buffered saline (PBS). The brains were then dissected and processed for Aβ immunostaining.

### Serial Differential Fractionation with Ultracentrifugation

To detect differences in the level of APP metabolic products among treatment groups, the levels of Aβ, Fl-APP, pAPPThr668, CTFs, sAPPα and sAPPβ-sw were measured after a 3-step sequential extraction using Tris buffered saline (TBS) (pH 7.4), 1% Triton X-100/TBS (TBSX) and formic acid (FA) methods as described elsewhere [26]. All extraction buffers also contained protease and phosphatase inhibitor cocktail (Roche Diagnostics, Basel, Switzerland). Snap frozen tissue was homogenized in 15 volumes (w/v) of TBS homogenization buffer and centrifuged at 100,000×g for 1 h at 4°C using a Type 70 Ti rotor in an Optima™ L-80XP Ultracentrifuge (Beckman Coulter, FL, USA). The TBS-soluble fraction was aliquoted, frozen in liquid nitrogen and stored at −80°C. The pellets were resuspended in 15 volumes of TBSX and kept on ice for 30 min, followed by a second centrifugation at 100,000×g for 1 h at 4°C. The TBSX soluble fraction was aliquoted, frozen and stored as for the TBS fraction. The TBSX-insoluble pellet was resuspended in 2 mL of 70% FA and centrifuged at 100,000×g at 4°C for 1 h. The FA extracts were neutralized by the addition of 20 volumes of 1 M Tris pH 11, aliquoted and stored at −80°C. Total protein content in TBS- and TBSX-extracts was determined via BCA assay. Total protein in FA-extracts was determined by Bradford Protein Microassay (Bio-Rad, CA, USA).

### Aβ Measurements

The levels of intracellular Aβ1–40 and Aβ1–42 were quantified by using a sandwich ELISA as previously described by Durairajan et al. [15],[24], with minor modification. Equal amounts of cell or brain lysates were used for Aβ quantification by sandwich ELISA. The monoclonal antibody 6E10 was used as the capture antibody by adding to ELISA plates (0.2 μg diluted in 0.1 M Na_2_CO_3_ (pH 9.6) per well) and incubated overnight at 4°C. The plates were washed with PBS (0.05% Tween 20) and blocked with 4% BlockAce (Serotec, Raleigh, NC, USA) for 2 h at room temperature. Equilibrated protein lysates from each treatment (a 100 μL volume was adjusted in all treatment groups by using PBS) were applied in duplicate and incubated at room temperature for 2 h with constant rotation at 30 rpm. Biotinylated monoclonal anti-Aβ40 5C3 (50 ng per well) and biotinylated monoclonal 8G7 (50 ng per well) antibodies were used for detection of Aβ1–40 and Aβ1–42, respectively, and were diluted in 1% BlockAce and incubated for 2 h at room temperature. Plates were thoroughly washed with PBS (0.05% Tween 20), and streptavidin conjugated HRP was added for 1 h at room temperature. Finally the plates were washed four times with PBST before adding the substrate TMB for 30 min. Absorbance at 450 nm was measured in duplicate wells after addition of 2 M H_2_SO_4_. All ELISA experimental data were from three different days. Synthetic Aβ40 and Aβ42 peptides were used for construction of calibration curves, and Aβ was measured in lysates.

### SDS-PAGE and Western Blot Analysis

For Western blot analysis from cell culture or mouse brain, 10–30 μg total protein was separated on 10% and 15% SDS–PAGE gels and blotted onto PVDF membranes for the detection of full length APP (Fl-APP), CTFs, sAPPα, sAPPβ-sw, β-actin and phosphorylated APP (pAPPThr668). After blocking with 5% skim milk, the blots were incubated with primary antibodies overnight at 4°C with shaking. Blots were washed and incubated with HRP-conjugated secondary antibodies. The goat anti-mouse IgG was used when the primary antibody was 6E10 (1∶1000), sAPPβ-sw (1∶100), or anti-β-actin (1∶5000), and the goat anti-rabbit IgG was used when the primary antibody was anti-CT15 (1∶5000) or pAPPThr668 (1∶1000). After incubation with HRP-conjugated secondary antibody, immunoblots were treated with ECL and developed using X-ray films (Fujifilm). Films were scanned, and the band intensity was analyzed using Image J software (NIH Image).

Membranes probed with primary antibodies CT15 and 6E10 was stripped using stripping buffer (62.5 mM Tris–HCl pH 7.6, 100 mM 2-mercaptoethanol and 2% SDS) at 60°C for 20 min, then washed with a generous amount of TBST for 20 mins twice and finally blocked with 5% milk for 1 h. Stripped CT15 and 6E10 probed membranes were reincubated with pAPPThr668 and sAPPβ-sw 6A1 antibodies, respectively.

### Immunohistochemistry

Immunohistochemistry and image analysis of Aβ plaques was performed on coronal brain sections from TgCRND8 mice treated with baicalein or tap water as described previously by Durairajan et al. [15]. For immunohistochemical analysis, 30 μm thick sections were obtained using a Thermo Shandon Cryotome® (Thermo Sceintific) slicing system. The free-floating sections were quenched for the endogenous peroxidase activity, and the sections were incubated overnight at 4°C with a biotinylated4G8 antibody (1∶1000);. After removing excess primary antibody, sections were washed 3 times, and immunostaining was performed using a Vectastain ABC Elite kit (Vector Laboratories, Burlingame, CA, USA) linked with the diaminobenzidine reaction. Images were obtained with a Nikon fluorescent inverted microscope with digital Nikon camera and analyzed by Image J software. Aβ plaque burden was calculated as the area occupied by the Aβ plaques as a percentage of total area of the brain sections.

### Statistical Analysis

The results are displayed as mean ± standard error (SE), with *n* = 3 or 5 per group for all comparisons. Statistical analysis was performed by one-way analysis of variance (ANOVA) followed by Fisher's Least Significant Difference (LSD) for *in vitro* experiments. In animal experiments, the student T test was performed. Statistical significance was accepted at *p<0.05, **p<0.01 and ***p<0.001.

## Results

### Cell Viability

**Figure 2:**
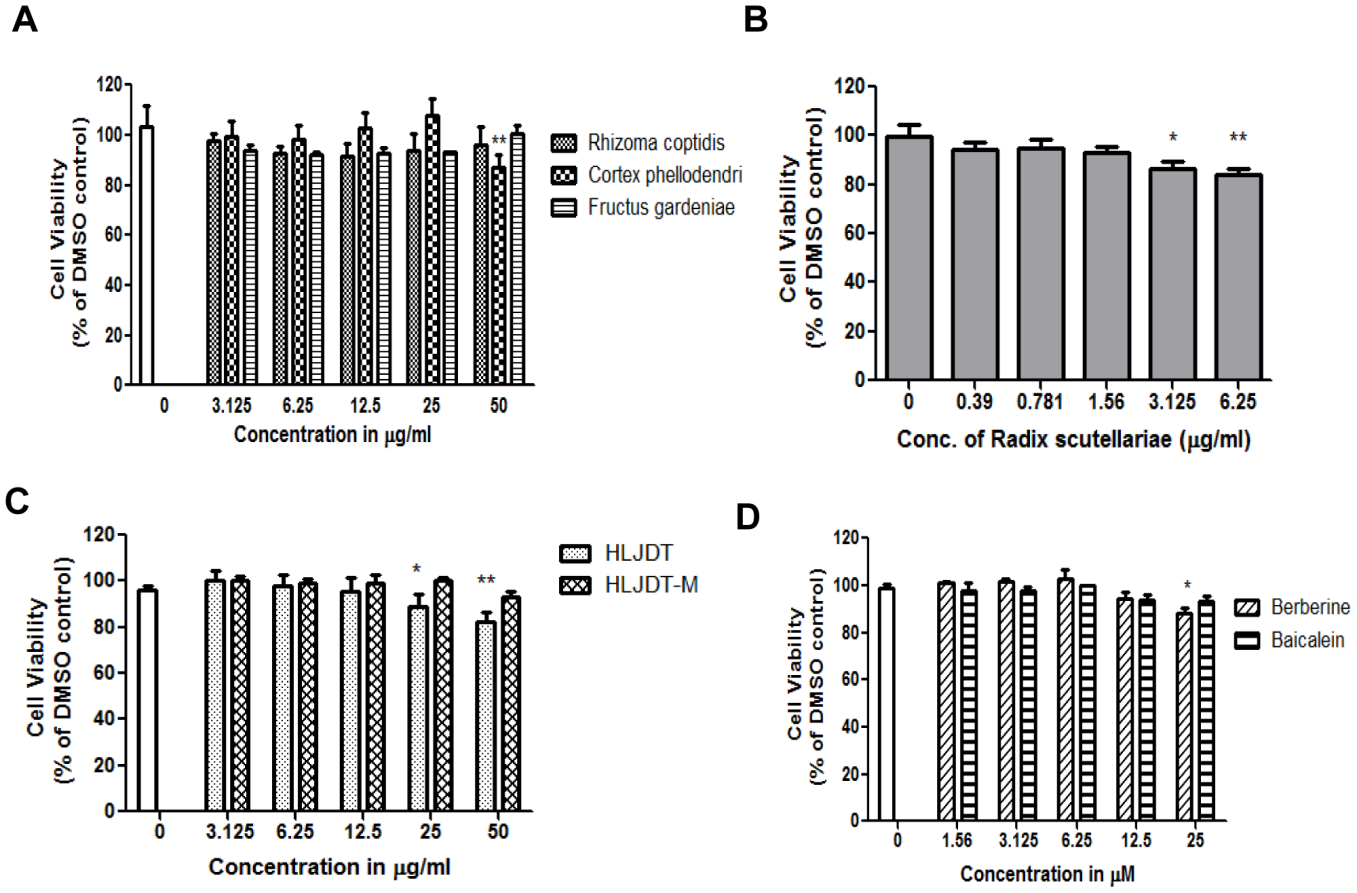
Effects of HLJDT, HLJDT-M, constituent herbs and constituent compounds on toxicity in N2a-SwedAPP cells. N2a-SwedAPP cells were treated with the ethanolic extracts of (A) RC, CP, FG, or (B) RS; (C) HLJDT or HLJDT-M; or (D) berberine or baicalein. Cell viability was determined by MTT assay. The values denote the results of three different experiments and are shown as mean±S.E.M. P values are for comparison with control: *p<0.05, **p<0.01.

N2a-SwedishAPP cells are widely used as a cellular model of Alzheimer’s disease, because they express a high level of APP and Aβ [23]. The effects of HLJDT and its components on viability of N2SwedAPP cells were monitored by using the MTT assay. We ascertained each extract and compound of HLJDT components for cytotoxicity for at least 48 h at five different concentrations; we considered the non-toxic concentration as the highest concentration that showed more than 90% cell viability (Figure 2). The DMSO concentration is 0.1% throughout the experiments and there is no cytotoxicity at this concentration. The non-toxic concentrations of RC (50 μg/mL), CP (25 μg/mL), FG (50 μg/mL), RS (1.56 μg/mL), HLJDT (12.5 μg/mL) HLJDT-M (25 μg/mL), berberine (12.5 μM) and baicalein (12.5 μM) were used to investigate the APP modulating effects on N2SwedAPP cells for 48 h. According to the contents of pure compounds in each herbal component of HLJDT (Table S1), there is a correlation between the effective concentration of berberine in RC (11 μM of berberine in 25 μg/mL of RC) and berberine (12.5 μM) alone, however, there is no exact correspondence between the highest effective concentration of baicalien in RS (0.1 μM of baicalein in 1.56 μg/mL of RS) and baicaein (12.5 μM) alone. The non-toxic concentrations of HLJDT components were used in the following experiments.

### Modulation of APP Processing by RC, RS, CP, and FG and S

We used Western blotting of N2a-SwedAPP cells to test for differences in the processing of Swedish mutant APP by HLJDT components. We used several antibodies in Western blot analysis: CT15 recognizes full length APP (Fl-APP), 6E10 identifies only α-secretase-cleaved APP i.e sAPPα, and the sAPPβ-sw specific for β-secretase cleavage of Swedish mutant APP. Several studies suggest that APP phosphorylation affects the maturation and subcellular distribution of APP, increases production of CTFs, and stimulates generation of Aβ [26],[27], therefore we examined APP phosphorylated at threonine 668 using an antibody to pAPPThr688.

**Figure 3:**
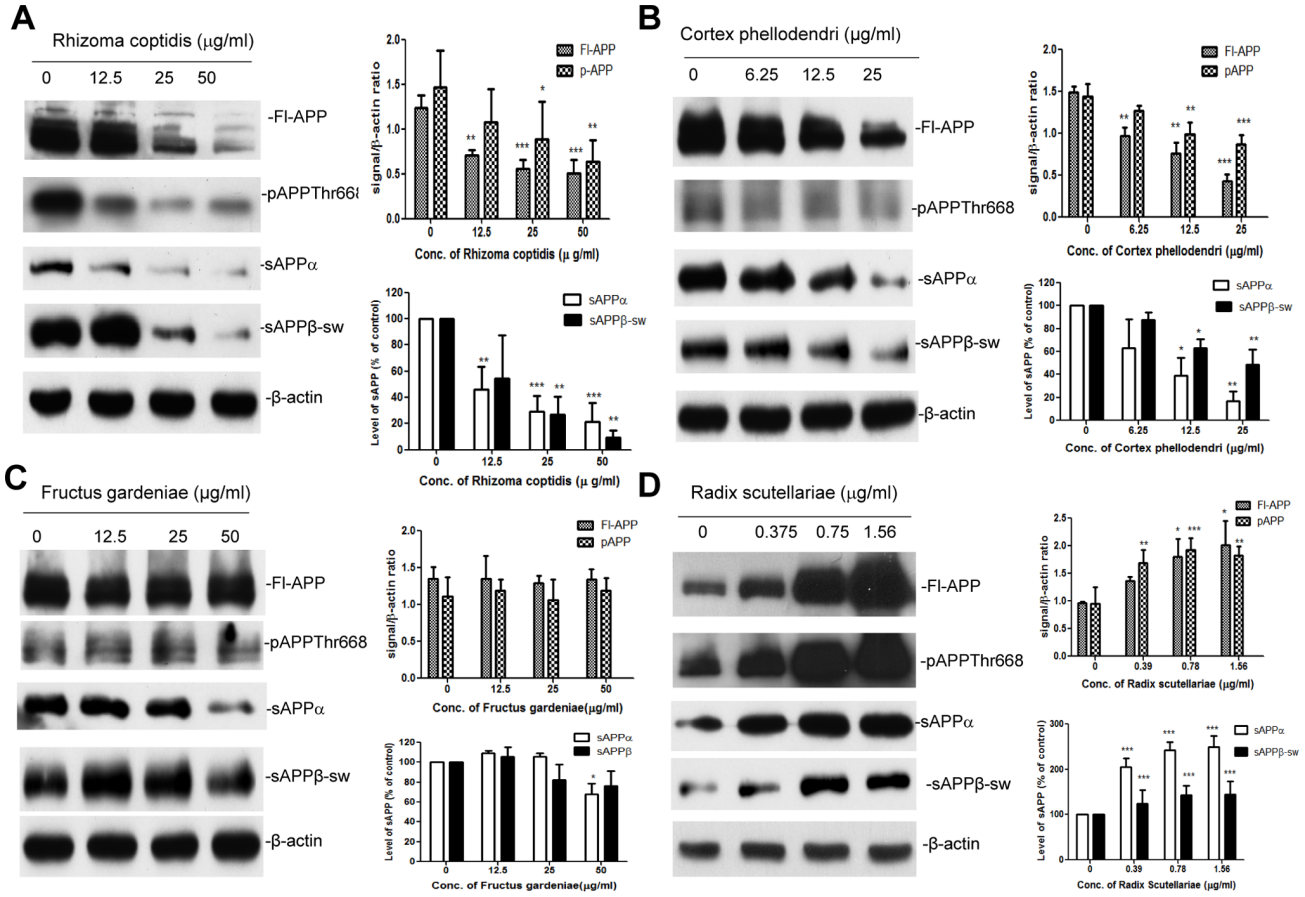
Extracts of HLJDT constituent herbs alter the processing of APP in N2a-SwedAPP cells. Conditioned medium and cell lysates were prepared from N2a-SwedAPP cells that were treated with (A) RC, (B) CP, (C) FG or (D) RS at various doses as indicated for 48 h. Western blotting was used to detect sAPPα and sAPPβ-sw in conditioned medium, and to detect Fl-APP, pAPPThr668 and β-actin in cell lysates. Bars represent mean±S.E.M. for three experiments. One-way ANOVA revealed significant differences due to treatment at various doses of components of HLJDT: *p<0.05; **p<0.01; ***p<0.001.

As shown in Figure 3, the levels of Fl-APP, pAPPThr688 and soluble APPs (sAPPα and sAPPβ-sw) were decreased by treatment with RC or CP in a concentration-dependent manner (Figure 3A and 3B). A low concentration of RC or CP (12.5 μg/mL) reduced the Fl-APP level by 42% or 38%, respectively. At 25 μg/mL, Fl-APP was reduced by 55% or 71% by RC or CP, respectively. Treatment with RC or CP decreased the level of phosphorylated APP. At 25 μg/mL, pAPPThr668 was reduced by 39.5% or 40.5% by RC or CP, respectively. Thus, RC or CP reduced the levels of Fl-APP and pAPPThr668. There was also a clear decrease in the levels of sAPPα and sAPPβ-sw. The level of sAPPα was reduced by 71% (p<0.001) or 83% (p<0.01) by treatment with RC or CP, respectively, at a concentration of 25 μg/mL. Similarly the level of sAPPβ-sw dropped significantly upon treatment with 25 μg/mL (p<0.01) of RC or CP, but 12.5 μg/mL of RC had no significant effect (p>0.05).

However, FG did not influence the level of Fl-APP, nor did it affect pAPPThr668 metabolism; the only significant effect of FG was a slight decrease in sAPPα at a concentration of 50 μg/mL (Figure 3C). In contrast with RC, CP and FG, RS significantly increased soluble APPs, intracellular APP and pAPPThr668 in a dose-dependent manner (Figure 3D). The level of maximal stimulation of Fl-APP and pAPPThr668 by RS is 1.89- and 2-fold of basal release, respectively, at a concentration of 1.56 μg/mL. The release of sAPPs was accelerated by treatment with RS in a dose dependent manner, reaching maximal augmentation at 1.4- and 2.4- fold of basal release for sAPPβ-sw and sAPPα, respectively, at a concentration of 1.56 μg/mL. These results demonstrate that the herbal components of HLJDT show differential modulation of APP processing.

### Modulation of APP Processing by HLJDT and HLJDT-M

**Figure 4:**
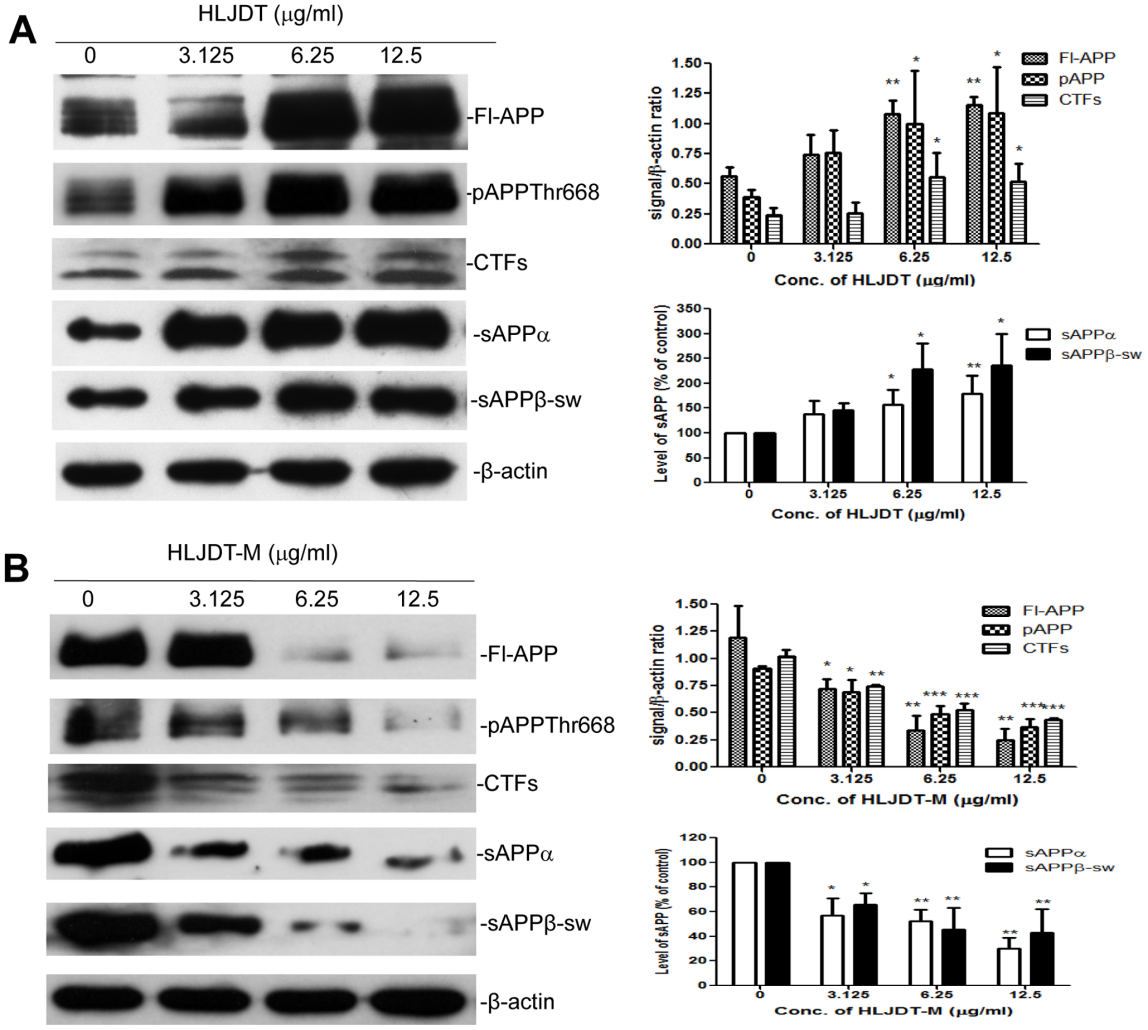
Modulation of APP processing by HLJDT and HLJDT-M in N2a-SwedAPPcells. Conditioned medium and cell lysates were prepared from N2a-SwedAPP cells that were treated with (A) HLJDT or (B) HLJDT-M at various doses as indicated for 48 h. Western blotting was used to detect sAPPα and sAPPβ-sw in conditioned medium, and to detect Fl-APP, pAPPThr668, CTFs and β-actin in cell lysates. Bars represent mean±S.E.M. for three experiments. One-way ANOVA revealed significant differences due to treatment at various doses of HLJDT or HLJDT-M: *p<0.05; **p<0.01; ***p<0.001.

Though RS increased APP metabolism, we still sought to test the effect of HLJDT on APP metabolism in N2aSwedAPP cells because we speculated that the other herbal components in the formula might lead to a net reduction in APP metabolism. In contrast to our expectation, HLJDT significantly increased soluble APPs, intracellular APP, pAPPThr668 and CTFs in a dose-dependent manner (Figure 4A). The level of maximal increase of Fl-APP and pAPPThr668 by HLJDT was 2.0- and 2.8- fold of basal release, respectively, at a concentration of 12.5 μg/mL. HLJDT treatment not only increased the level of Fl-APP and pAPPThr668 but also dose-dependently increased the generation of CTFs, confirming the APP-increasing effect of HLJDT. HLJDT increased CTFs to 2.1 or 2.3 times the basal levels at concentrations of 12.5 (p<0.05) or 6.25 (p<0.05) μg/mL, respectively. Accordingly, sAPPα and sAPPβ-sw were also elevated. The release of sAPPs was accelerated by treatment with HLJDT in a dose-dependent manner, reaching maximal augmentation at 1.4 and 2.4 fold of basal release for sAPPβ-sw and sAPPα, respectively, at a concentration of 3.125 μg/mL. The increase in sAPPs levels is consistent with the increase in Fl-APP and CTFs levels by HLJDT, and indicates that HLJDT increases amyloidogenic processing of APP.

Since both RS and HLJDT increased APP metabolism, we modified the HLJDT formula (HLJDT-M) by removing RS; RC, CP and FG were included in HLJDT-M at a ratio of 4∶24, respectively. As expected, HLJDT-M significantly and dose-dependently decreased all metabolic products of APP to an even greater extent than RC and CP alone. HLJDT-M treatment at a concentration of 12.5 μg/mL reduced the levels of Fl-APP, pAPPThr668 and CTFs by 79% (p<0.01), 60% (p<0.001) and 68% (p<0.001), respectively (Figure 4B). The levels of sAPPα and sAPPβ-sw were reduced by 70% (p<0.001) and 57% (p<0.01), respectively (Figure 4B). The removal of RS from HLJDT totally reversed the amyloidogenic property of HLJDT.

### Modulation of APP Processing by Berberine and Baicalein

**Figure 5:**
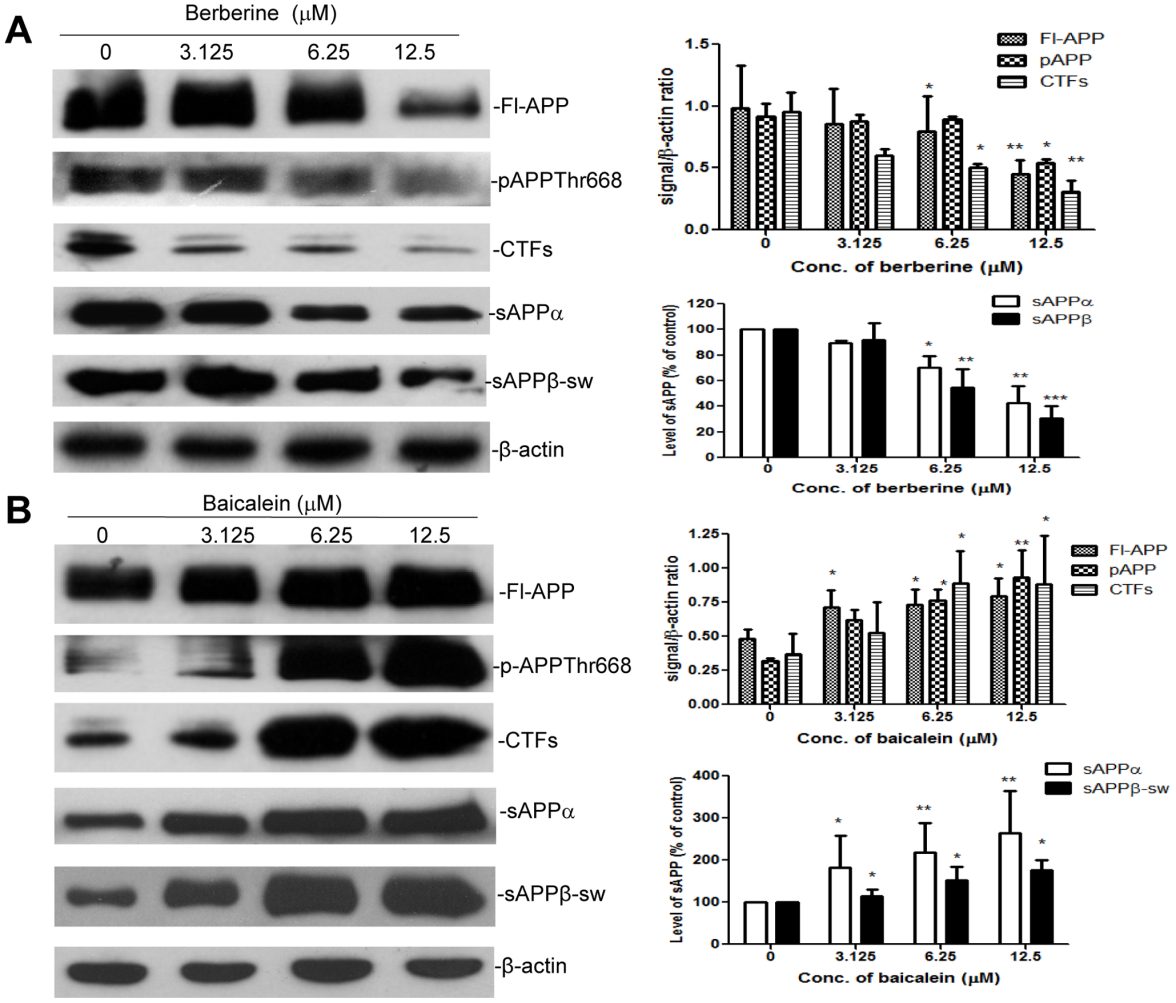
Modulation of APP processing by berberine and baicalein in N2a-SwedAPP cells. Conditioned medium and cell lysates were prepared from N2a-SwedAPP cells that were treated with (A) berberine or (B) baicalein at various doses as indicated for 48 h. Western blotting was used to detect sAPPα and sAPPβ-sw in conditioned medium, and to detect Fl-APP, pAPPThr668, CTFs and β-actin in cell lysates. Bars represent mean±S.E.M. for three experiments. One-way ANOVA revealed significant differences due to treatment at various doses of berberine or baicalein: *p<0.05; **p<0.01; ***p<0.001.

Berberine and baicalein have been extensively studied as markers for HLJDT, therefore the effects of these pure components of HLJDT on APP metabolism were examined. We had already determined that berberine significantly decreases Aβ, pAPPThr688 and CTFs in both *in vivo* and *in vitro* models of AD [15]. However, we had not studied the effect of berberine on the level of soluble APPs; therefore we proceeded to assess the effect of berberine on APP metabolism in N2a-SwedAPP cells. As expected, berberine significantly decreased soluble APPs, intracellular APP, pAPPThr668 and CTFs in a dose-dependent manner (Figure 5A). Levels of sAPPα and sAPPβ-sw were reduced by 58% (p<0.01) and 70% (p<0.001), respectively, by treatment with 12.5 μM berberine, and by 30% (p<0.05) and 45% (p<0.01) by 6.25 μM berberine. Berberine treatment at a concentration of 12.5 μM reduced the level of Fl-APP, pAPPThr668 and CTFs by 54% (p<0.01), 42% (p<0.05) and 67% (p<0.01), respectively. Since RS and HLJDT increased all APP metabolic products, we ascertained whether baicalein alone can induce the same APP increasing effect as did RS and HLJDT. As hypothesized, baicalein significantly increased soluble APPs, Fl-APP, pAPPThr668 and CTFs in a dose-dependent manner (Figure 5B). The levels of maximal increase of Fl-APP, pAPPThr668 and CTFs by baicalein were 1.68 (p<0.05), 1.58 (p<0.01) and 2.36 (p<0.05) fold of basal release, respectively, at a concentration of 12.5 μM. The release of sAPPs was accelerated by treatment with baicalein in a dose-dependent manner, reaching maximal secretion of 2.65 (p<0.01) and 1.70 (p<0.05) fold of basal release for sAPPα and sAPPβ-sw, respectively, at a concentration of 12.5 μM. These data suggest that berberine is one of the alkaloids responsible for the APP-decreasing effect of HLJDT-M, and baiclein is one of the flavonoids responsible for the APP-increasing effect of HLJDT.

### Modulation of Intracellular Aβ Levels by HLJDT, HLJDT-M and its Components in N2a-SwedAPP Cells

**Figure 6:**
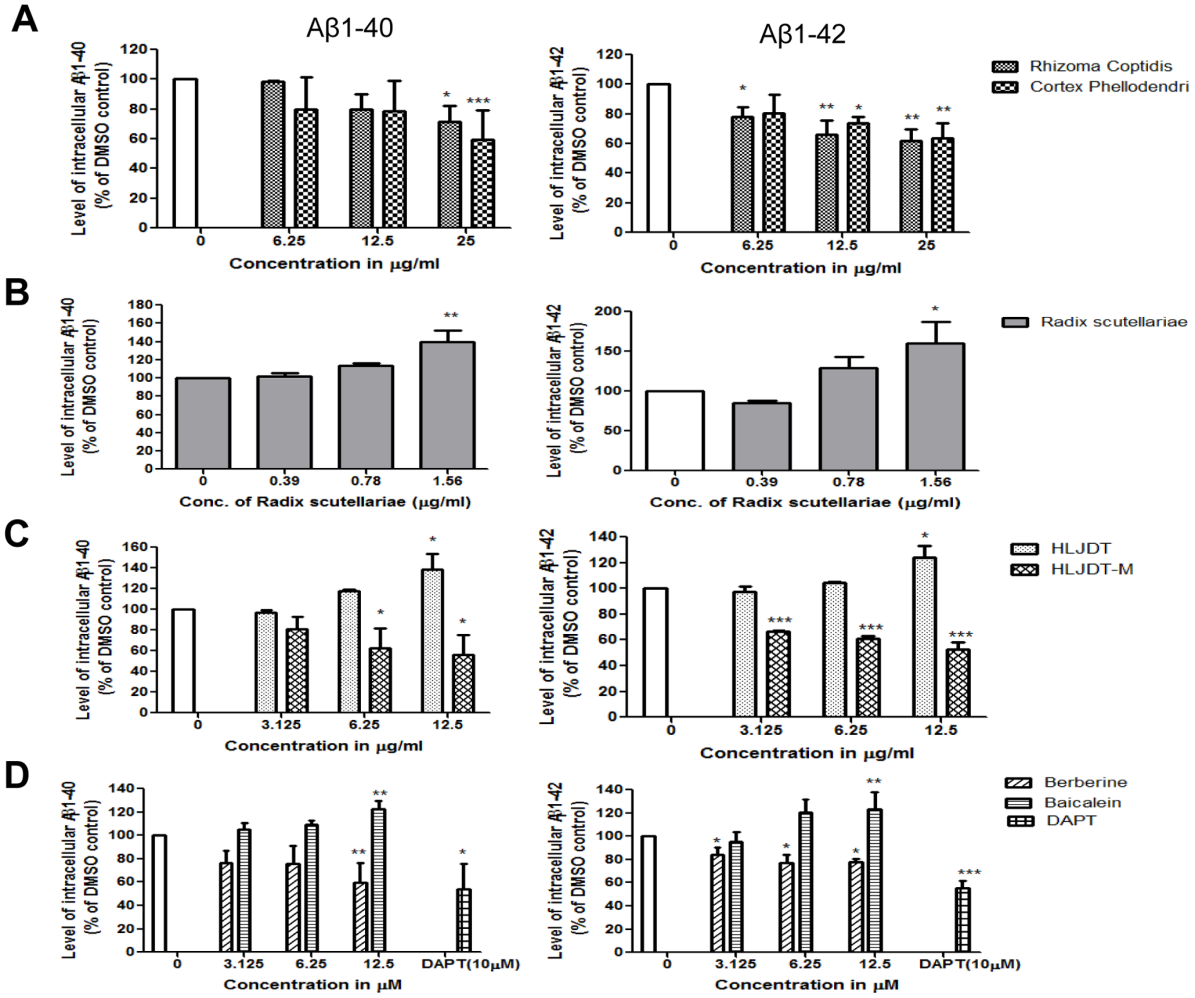
Regulation of the levels of intracellular Aβ by HLJDT, HLJDT-M and its components in cultured N2a-SwedAPP cells. Aβ1–40 and Aβ1–42 peptides were analyzed by enzyme-linked immunosorbent assay (ELISA) in N2a-SwedAPP cell lysates 48 h after addition of (A) RC or CP; (B) RS; (C) HLJDT or HLJDT-M; or (D) berberine or baicalein. Bars represent mean±S.E.M. of the level of intracellular Aβ1–40 or Aβ1–42 peptides in three experiments relative to DMSO control (untreated). One-way ANOVA revealed significant differences due to treatment: *p<0.05; **p<0.01; ***p<0.001.

Since HLJDT and its components modulated intracellular levels of APP and CTFs in N2a-SwedAPP, we investigated the effect of HLJDT and its components on the level of intracellular Aβ, which is a key factor in AD progression [29]. We treated N2a-SwedAPP cells with different non-toxic concentrations of HLJDT, HLJDT-M and its components and then analyzed intracellular Aβ levels by ELISA (Figure 6). There was a dose-dependent reduction in both Aβ1–40 and Aβ1–42 by both RC and CP treatments. Intracellular Aβ1–40 markedly dropped 41% and 29% due to treatment by 25 μg/mL RC and CP, respectively (Figure 6A). There was a 32% reduction in intracellular Aβ1–42 in both RC and CP treated cells at the same high concentration. In contrast, RS treatment increased intracellular Aβs in a dose-dependent manner, reaching maximal accumulation of 1.4- and 1.6- fold of basal levels of Aβ1–40 and Aβ1–42, respectively, at a concentration of 1.56 μg/mL (Figure 6B).

When comparing the effects of HLJDT and HLJDT-M on the level of intracellular Aβs, as expected, HLJDT increased both Aβ1–40 and Aβ1–42 in a dose-dependent manner, reaching maximal accumulation of 1.4- and 1.6- fold of basal level, respectively, at a concentration of 12 μg/mL (Figure 6C). By contrast, HLJDT-M decreased both Aβ1–40 and Aβ1–42 in a dose-dependent manner. Intracellular Aβ1–40 markedly dropped 44% with 12.5 μg/mL of HLJDT-M, 38% with 6.25 μg/mL and 20% with 3.12 μg/mL. Aβ1–42 was reduced 48% by 12.5 μg/mL of HLJDT-M, 39% with 6.25 μg/mL and 33% with 3.12 μg/mL (Figure 6C). These data suggest that HLJDT-M, which lacks RS, significantly reduced intracellular Aβs, and that the Aβ-reducing effect is far better than that of the individual herbal components of HLJDT-M. We also confirmed the above outcome by using two pure HLJDT components (berberine and baicalein). At a concentration of 12.5 μM, berberine significantly reduced both Aβ1–40 and Aβ1–42 by 37% and 25%, respectively, whereas baicalein significantly augmented both Aβ1–40 and Aβ1–42 to 1.23 and 1.38 fold of basal level (Figure 6D). These findings provide evidence regarding the effects of HLJDT, HLJDT-M and its components on APP metabolism and Aβ levels.

### Chronic Baicalein Administration Increases Aβ Plaque Burden, Aβ Levels and Amyloidogenic APP Processing in TgCRND8 Mice

**Figure 7:**
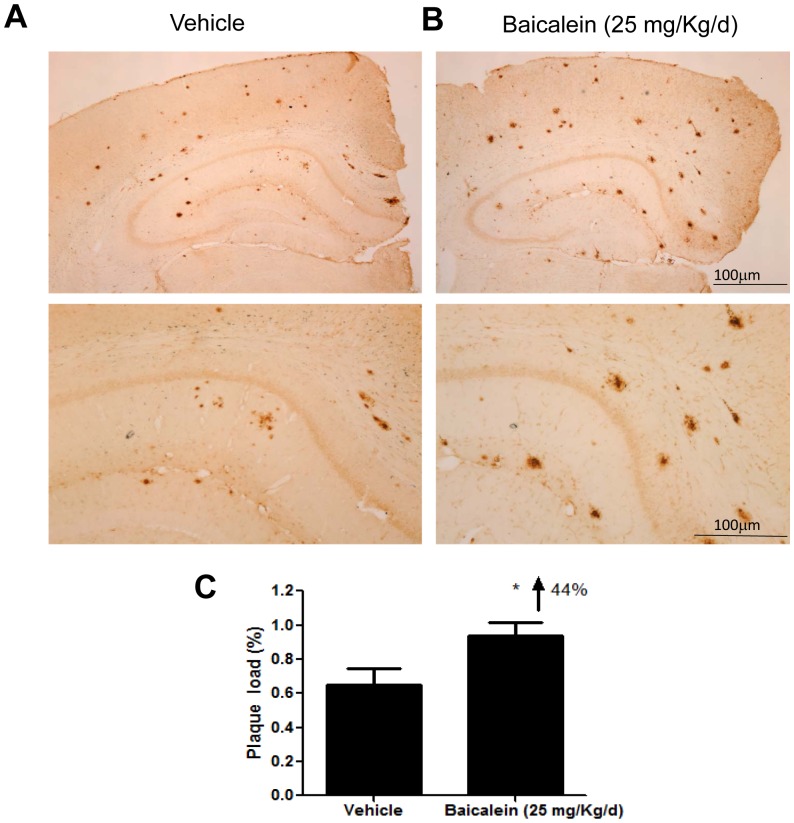
Baicalein increased cortico-hippocampal Aβ plaque pathology in TgCRND8 mice. Coronal sections of TgCRND8 mice treated with (A) vehicle or (B) baicalein (25 mg/kg per day) and sacrificed after 3 months of treatment, followed by immunohistochemical staining for Aβ using 4G8 antibody. Digital images of cortex and hippocampus were captured and analyzed with Image J software. Scale bar: 100 μm. (C) The percentage of coronal brain area occupied by Aβ immunoreactivity. Bars represent mean±S.E.M. for five mice per group. Student’s t-test revealed a significant difference due to treatment: *p<0.05.

Since cell models of AD may not represent the *in vivo* efficacy of herbal components, we have tested the efficacy of berberine and baicalein, two widely studied compounds in HLJDT, in a TgCRND8 mouse model of AD. We recently reported that chronic administration of berberine can significantly reduce Aβ pathology, gliosis and cognitive impairments in TgCRND8 mice via reducing the level of C-terminal fragments of APP and the level of phosphorylated APP [15]. However the observation that baicalein treatment increased APP metabolites and intracellular Aβ in a cell culture study directed us to examine the effect of baicalein on Aβ plaque load in TgCRND8 mice. We found that chronic oral administration of baicalein at 25 mg/kg per day for nearly 3 months did not significantly change animal body weight, nor did it cause any notable adverse side effects in TgCRND8 mice (data not shown). Immunostaining of amyloid plaques in the brains of TgCRND8 mice revealed that baicalein treatment increased the area occupied by Aβ deposits in cortex and hippocampus by 44% (p<0.05) (Figure 7).

**Figure 8:**
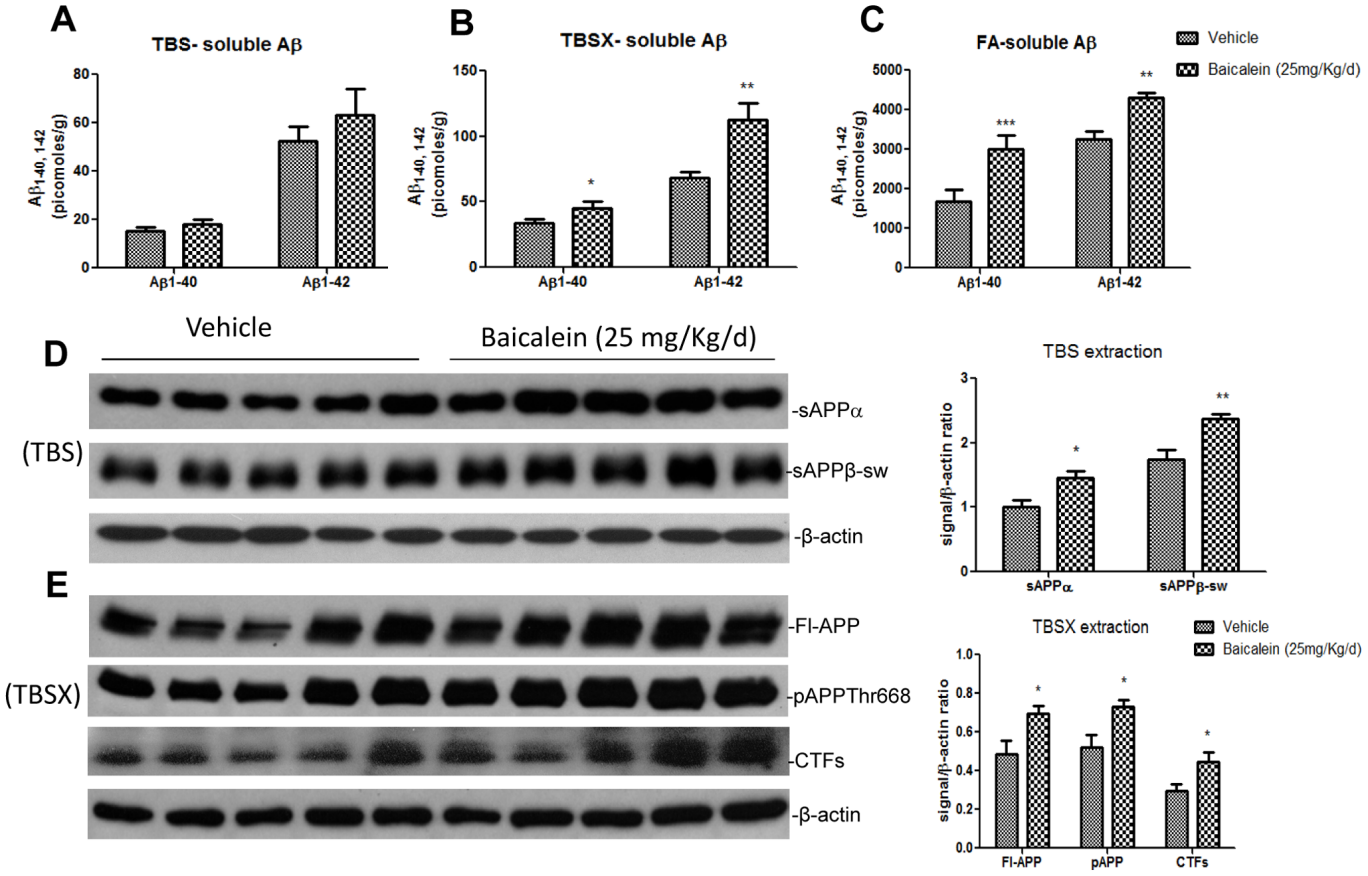
Baicalein treatment increases amyloidogenic processing in TgCRND8 mice. Serial extraction of (A) TBS-soluble, (B) TBSX-soluble (C) and FA-soluble Aβ1–40 and Aβ1–42 from cerebral hemispheres of five vehicle-treated or five baicalein-treated (25 mg/kg per day) TgCRND8 mice, measured by sandwich ELISA. Immunoblots demonstrating the levels of sAPPs, Fl-APP, pAPPThr668, CTFs and β-actin in (D) TBS- and (E) TBSX-extracted brain lysates from the above mice. Densitometric analysis of the immunoblots, performed using Image J, with signals normalized to those for β-actin. Bars represent mean±S.E.M. for five mice per group. Student’s t-test revealed a significant difference due to treatment: *p<0.05, **p<0.01.

The above results of increased 4G8-positive Aβ deposits by baicalein treatment were further ascertained by Aβ ELISA analysis in the other hemisphere of the brain. Aβ1–40 and Aβ1–42 levels in TBS, TBSX and FA brain fractions were measured via ELISA. Baicalein treatment did not significantly affect TBS soluble Aβ levels but did significantly increase both TBSX-soluble and insoluble formic acid fractions (Figure 8A, 8B and 8C). In the TBSX-soluble fraction, the increases of Aβ1–40 and 1–42 were 35% (p<0.05) and 65% (p<0.01), respectively, whereas in the FA fraction, the corresponding increases were 80% (p<0.001) and 32% (p<0.01).

To examine the modulation of APP processing by baicalein treatment, we subjected the serially extracted fractions to Western blot analyses to specifically detect soluble and membrane associated APP as described elsewhere. The TBS fraction was analyzed for sAPPα and sAPPβ-sw, and the TBSX fraction was analyzed for Fl-APP, CTFs and pAPPThr668. Antibodies specific for sAPPβ-sw and sAPPα revealed increases in signal intensities of 35% (p<0.01) and 44% (p<0.05), respectively, after baicalein treatment (Figure 8D). Immunoblot analysis also showed 30%, 40% and 41% increases in the levels of Fl-APP, CTFs, and pAPP, respectively (Figure 8D). The increased metabolic products of APP following baicalein treatment in mice is also consistent with our interpretation of *in vitro* data that baicalein increases amyloidogenic processing of APP.

## Discussion

The clinical uses of HLJDT, which has been traditionally prescribed in China to treat gastrointestinal diseases, acute liver injury and cardiovascular diseases [30],[31], have now been extended to treat patients with cerebrovascular disease and vascular dementia in China and Japan [6],[7],[8]. An improved formula of HLJDT in a pill form has been approved by the Chinese State Food and Drug Administration (drug approval number Z20025356) [32]; however, the approval by the regulatory authorities for use in AD dementia in both China and Japan is still pending due to lack of efficacy data. Pharmacological *in vitro* and *in vivo* studies have confirmed the neuroprotective activities of HLJDT against cerebrovascular diseases [10],[13],[14]. Three single pure compounds (berberine, baicalein and geniposide) from HLJDT show activities in different AD models [15],[19],[20]. In an experimental plan primarily designed to identify key herbs and compounds responsible for the neuroprotective effects of HLJDT in an *in vitro* AD model, N2aSwedAPP cells, we unexpectedly found that RS alone and HLJDT instead strongly increased Fl-APP, pAPPThr668, CTFs, sAPPs and Aβ (Figure 3D and 4A). The increased Aβ levels also translated into increased amyloid plaque burden in a mouse model of Alzheimer’s disease (Figure 7 and 8). However, the modified HLJDT (that is HLJDT-M, which is HLJDT without RS) significantly decreased all the APP metabolic products including Aβ (Figure 4B and 6C). HLJDT is a formulation of RC, RS, CP and FG at the ratio of 3∶2:2∶3. However, the herbal components of HLJDT-M (RC, CP, FG) were prepared at the ratio of 4∶2:4 respectively due to the following reasons: 1) We found that RS enhances Aβ load and thus reduces the efficacy of the HLJDT formula; 2) Since RS is an anti-oxidative herb [33],[34] and its removal from HLJDT-M may reduce the anti-oxidative capacity of HLJDT-M; and, 3) Because FG and its pure compounds have been shown to have significant antioxidative and memory enhancing activity without influencing APP modulation [35],[36], we have increased 3 parts FG to 4 parts; 4) Because RC is considered to be the “King drug” of HLJDT, and the amounts of related protoberberine alkaloids (e.g. palmatine) in RC are significantly higher compared to the adjunctive drug CP (Table S1), we have increased 3 parts RC to 4 parts; and 5) In order to bring the total to 100%, the amount of CP was unaltered, at 2 parts. Since we have recently determined that berberine can reduce pAPPThr668, CTFs and amyloid plaque burden in a mouse model of AD [15], we conclude that the Aβ-reducing effect of RC, CP or HLJDT-M may be mainly due to the presence of berberine in HLJDT-M. We found that berberine can decrease both sAPPα and sAPPβ-sw, in addition to reducing pAPPTh668 and CTFs accumulation (Figure 5A and 6D). Since sAPPα and sAPPβ-sw have recently been considered potential markers of AD [2], the sAPPs reducing effect of berberine adds to the evidence for its anti-AD effect. HLJDT-M shows more significant APP- and Aβ- reducing effects than berberine, RC or CP treatment alone. The greater APP and Aβ reducing effect of HLJDT-M is most probably due to the synergistic action of related protoberberine alkaloids (coptisine, palmatine, jatterorhizine etc.) with berberine. Similarly, the greater APP and Aβ increasing effect of RS or HLJDT is most probably due to the synergistic action of other flavonoids (baicalin, wogonin, wogonoside etc.) with baicalein. The overall influence of HLJDT components on APP metabolic products are shown in Table 1.

**Table 1:** The overall influence of HLJDT components on APP metabolic products.

Components	Fl-APP	pAPP	CTFs	sAPPα	sAPPβ	Aβs
RC	↓	↓	nd	↓	↓	↓
CP	↓	↓	nd	↓	↓	↓
FG	↔	↔	nd	↔	↔	nd
RS	↑	↑	nd	↑	↑	↑
HLJDT	↑	↑	↑	↑	↑	↑
HLJDT-M	↓	↓	↓	↓	↓	↓
Berberine	↓	↓	↓	↓	↓	↓
Baicalein	↑	↑	↑	↑	↑	↑

↓ decreased, ^↑^increased, ^↔^unchanged, ^nd^ not determined.

It has been revealed that APP phosphorylation at position Thr668 accelerates the accumulation of CTFs and increases Aβ generation [37]. Several studies have revealed that APP maturation and its targeting for proteolysis by secretases require APP Thr668 phosphorylation [27],[37]. The fast anterograde axonal transport of pAPPThr668 to the nerve terminals, where β- and α-secretase mediated cleavage occurs, results in increased Aβ release [27],[28],[37]. This indicates that berberine-mediated reduction of APP phosphorylation might subsequently result in a reduction of mature pAPPThr668 and a reduced level of Fl-APP engaged in distal axons of neurons, which would prevent Aβ generation at synaptic terminals. Our study on berberine is in line with a previous study showing that lithium and JNK inhibitor peptide (D-JNKI1) reduce the Aβ burden mainly via reducing CTFs, pAPP and Fl-APP [38],[39] without changing the level of BACE-1. In particular, D-JNKI1 has also been shown to reduce both sAPPα and β, similar to our finding that HLJTD-M, RC and their pure compound berberine significantly reduced sAPPs.

While there are several herbal compounds that have been found to reduce Aβ generation and its aggregation [15],[20],[24],[40],[41],[42],[43], baicalein is the first herbal compound that has been shown to potentiate Alzheimer’s pathology both *in vitro* and *in vivo* (Figure 5B, 6D, 7 and 8). Baicalein’s ability to increase APP, pAPPThr668 and CTFs as judged by Western analysis may partially account for the increased Aβ generation (Figure 5B, 6D, 7 and 8). A plausible mechanism by which baicalein enhances Aβ deposition is modulation of APP processing, because the levels of APP-CTFs, the direct precursor of Aβ [44], were increased by baicalein treatment (Figure 5b). APP processing can be modulated in a number of ways, such as alterations in the function of β-secretase (BACE-1) or variation in APP transport [45],[46],[47],[48]. We did not observe any significant change in the levels of BACE-1 protein (data not shown), thus baicalein may modulate APP processing through another mechanism. We believe that baicalein treatment augments the abnormal processing of neuronal APP generating amyloidogenic fragments that, upon proteolysis, form Aβ. Similar to our study on the Aβ potentiating effect of baicalein, the anticancer drug cladribine and the proton-pump inhibitor lansoprazole have recently been shown to significantly increase the generation of Aβ and amyloid plaques in cellular and animal models of AD [49], [50]. These Western drugs also significantly and dose dependently increased CTFs and sAPPs, in line with our findings that baicalein, RS and HLJDT mediated increases of APP metabolic products.

Since the completion of this manuscript, Zhang et al. [51] reported that baicalein reduces Aβ by promoting the non-amyloidogenic pathway in Chinese hamster ovary cells expressing wild type APP (CHO-APPwt) and in the Tg2576 mouse model of AD. This is in strong contrast to our reports that baicalein treatment of N2a-Swedish APP cells and of the TgCRND8 mouse model of AD resulted in a dramatic increase of APP and Aβ load. Although Zhang et al. showed that baicalein increases the secretion of sAPPα, they did not show corresponding increases in the CTFα level or decreases in the sAPPβ level. In fact, their data appeared to show an increase in the level of Fl-APP in CHO-APPwt cells although they claimed that the level of Fl-APP did not change, yet no details were provided regarding quantification of the level of Fl-APP. Another difference between our studies was the duration of incubation with baicalein. We treated cells with baicalein for 48 hours to observe the APP processing, while they treated only 12 hours. It is usually recommended that tests for the efficacy and toxicity of natural compounds should last 24–48 hours [52]. In addition, Zhang et al. did not perform immunohistochemistry of Tg2576 mouse brain sections to quantify the Aβ burden.

Although berberine and baicalein showed opposite effects on Aβ and are both components of HLJDT, the net effect of HLJDT was to increase Aβ load. Perhaps this is because baicalein (1–10 μM) has greater brain bioavailability [53] than berberine [15],[54]. Only one pharmacokinetic study has reported the concentration of baicalein (quantity not shown) in rat brain after oral administration of HLJDT; the concentration was very low after a dose of 20 g/kg HLJDT (equivalent to 30 mg/kg baicalein) [55]. In our study, the mean concentration of baicalein in the brain of TgCRND8 mice was 0.12±0.01 μg/mL when orally administered at a concentration of 25 mg/kg. In our *in vitro* study, the highest Aβ-increasing concentration of baicalein in HLJDT (12.5 μg/mL) was 0.16 μg/mL, which is comparable to the effective in vivo concentration of baicalein. These findings indicate that baicalein may induce amyloidogenic activity *in vivo*. Although HLJDT was shown to be neuroprotective with memory enhancing activity in the ischemic model [14], the APP-increasing effect of baicalein may surpass its memory enhancing activity in AD models. We consider that our study determines for the first time that baicalein can potentiate Aβ not only in a cell culture model but also in an animal model of AD. These findings can contribute to the design of further studies to assess whether treatment with HLJDT or HLJDT-M may have an influence on AD.

## Conclusions

In summary, these data provide convincing evidence for the first time that treatment with HLJDT or its components RS or baicalein can increase amyloidogenic processing of APP in a cell model of AD. In addition, chronic baicalein administration to TgCRND8 mice contributes to robustly increased plaque burden. Thus, chronic treatment with baicalein or RS or HLJDT may have deleterious effects in AD patients. However HLJDT-M (HLJDT without RS) may be suitable for AD therapy because of the presence of berberine and other protoberberine alkaloids, which may act synergistically to reduce Aβ production in AD patients.

## Supporting Information

Figure S1 **Typical base peak chromatogram (A) and extracted ion chromatograms (B) of HLJDT extracts.** 1. Geniposide; 2. Phellodendrine; 3. Columbamine; 4. Coptisine; 5. Epiberberine; 6. Jatrorrhizine; 7. Baicalin; 8. Palmatine; 9. Berberine; 10. Wogonoside; 11. Baicalein; and 12.Wogonin. Identity of peaks 1, 4, 6, 7, 8, 9, 11, and 12 were ascertained according to their m/z and retention time (Rt) when compared to 8 mixture reference solutions from the positive ion mode. The peaks 2, 3, 5, and 10 were identified based on published articles [21],[22] related to the profiling of chemical components from all the herbal components of HLJDT.
(TIF)

Table S1 **The contents (%) of geniposide, berberine, palmatine, baicalin, baicalein and wogonin in each herbal extract of HLJDT quantified by HPLC analysis.**
(DOCX)

Table S2 **The analytical condition of LC-MS for the identification of the 12 compounds detected in HLJDT by LC-MS.**
(DOCX)
