# Correction: Effects of Huanglian-Jie-Du-Tang and Its Modified Formula on the Modulation of Amyloid-β Precursor Protein Processing in Alzheimer's Disease Models

**DOI:** 10.1371/journal.pone.0102923

**Published:** 2014-07-11

**Authors:** The PLOS ONE Staff

There is an error in the sub-heading “Modulation of APP Processing by RC, RS, CP, and FG and S” under the “Results” section. The correct sub-heading for this section is:

“Modulation of APP Processing by RC, RS, CP, and FG”

Additionally, there is an error in the second paragraph of the sub-heading “Chromatographic Conditions” under the “Materials and Methods” section. There are no supplementary methods in the article, and Table S2 is incorrectly referenced as Table S1 in this paragraph. Therefore, the correct paragraph should read:

Qualitative identification of 12 active compounds of HLJDT was also performed by ultra-high performance liquid chromatography with quadrupole time-of-flight mass spectrometry (UHPLC-Q-TOF-MS) analysis with positive mode ESI-MS. A database was prepared for the identification of phytoconstituents of HLJDT based upon their molecular masses, calculated m/z values and retention times (Rt) observed in the analysis (Table S2).
