# Supplementary figures and images for: Effects of Huanglian-Jie-Du-Tang and Its Modified Formula on the Modulation of Amyloid-β Precursor Protein Processing in Alzheimer's Disease Models
Source: PLoS One. 2014 Mar 26;9(3):e92954. doi: 10.1371/journal.pone.0092954 (PMC3966845; doi:10.1371/journal.pone.0092954)

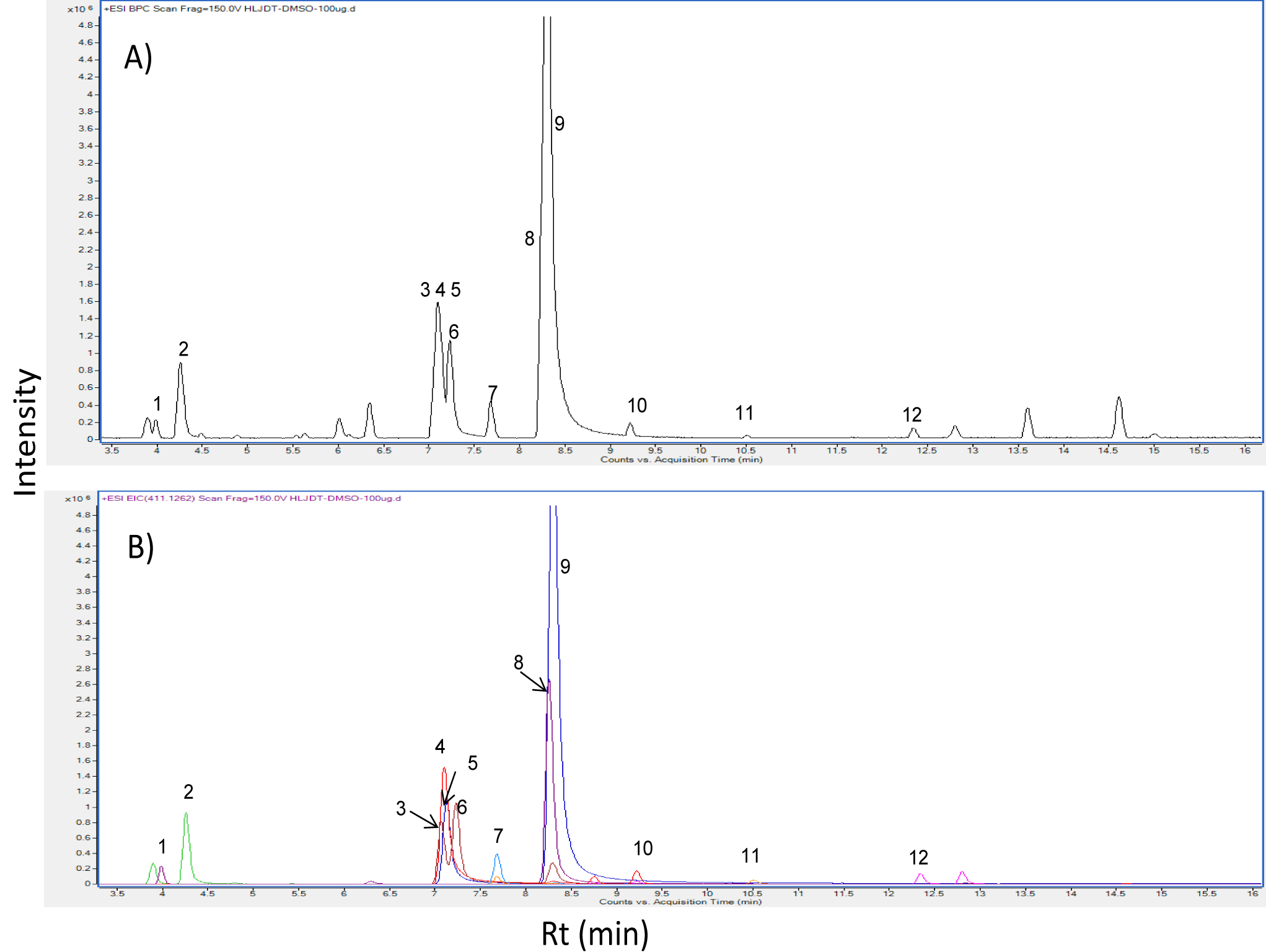

Supplement: Figure S1 — Typical base peak chromatogram (A) and extracted ion chromatograms (B) of HLJDT extracts. 1. Geniposide; 2. Phellodendrine; 3. Columbamine; 4. Coptisine; 5. Epiberberine; 6. Jatrorrhizine; 7. Baicalin; 8. Palmatine; 9. Berberine; 10. Wogonoside; 11. Baicalein; and 12.Wogonin. Identity of peaks 1, 4, 6, 7, 8, 9, 11, and 12 were ascertained according to their m/z and retention time (Rt) when compared to 8 mixture reference solutions from the positive ion mode. The peaks 2, 3, 5, and 10 were identified based on published articles [21],[22] related to the profiling of chemical components from all the herbal components of HLJDT. (TIF) [file pone.0092954.s001.tif]
