# Supplementary material for: Effects of Huanglian-Jie-Du-Tang and Its Modified Formula on the Modulation of Amyloid-β Precursor Protein Processing in Alzheimer's Disease Models
Source: PLoS One. 2014 Mar 26;9(3):e92954. doi: 10.1371/journal.pone.0092954 (PMC3966845; doi:10.1371/journal.pone.0092954)
Supplement: Table S1 — The contents (%) of geniposide, berberine, palmatine, baicalin, baicalein and wogonin in each herbal extract of HLJDT quantified by HPLC analysis. (DOCX) [file pone.0092954.s002.docx]

**Table S1**

The contents (%) of geniposide, berberine, palmatine, baicalin, baicalein and wogonin in each herbal extract of HLJDT quantified by HPLC analysis

| Components | Contents (%) | | | | |
| --- | --- | --- | --- | --- | --- |
|  | RC RS CP FG HLJDT | | | | |
| Geniposide | ／ | ／ | ／ | 19.44 | 4.01 |
| Baicalin | ／ | 5.57 | ／ | ／ | 2.67 |
| Palmatine | 2.69 | ／ | 0.409 | ／ | 0.867 |
| Berberine | 14.9 | ／ | 19.49 | ／ | 6.02 |
| Baicalein | ／ | 1.62 | ／ | ／ | 1.31 |
| Wogonin | ／ | 0.948 | ／ | ／ | 0.615 |

／ undetected
