# Supplementary material for: Effects of Huanglian-Jie-Du-Tang and Its Modified Formula on the Modulation of Amyloid-β Precursor Protein Processing in Alzheimer's Disease Models
Source: PLoS One. 2014 Mar 26;9(3):e92954. doi: 10.1371/journal.pone.0092954 (PMC3966845; doi:10.1371/journal.pone.0092954)
Supplement: Table S2 — The analytical condition of LC-MS for the identification of the 12 compounds detected in HLJDT by LC-MS. (DOCX) [file pone.0092954.s003.docx]

**Table S2**

The analytical condition of LC-MS for the identification of the 12 compounds detected in HLJDT by LC-MS

| Peak No. | Rt (min) | MS, m/z | Molecular formula | Identification |
| --- | --- | --- | --- | --- |
| 1 | 3.99 | 411.1262 [M+Na]^+^ | C_17_H_24_O_10_ | Geniposide |
| 2 | 4.29 | 342.1705 [M]^+^ | C_20_H_24_NO_4_ | Phellodendrine |
| 3. | 7.05 | 338.1392[M]^+^ | C_20_H_20_NO_4_ | Columbamine |
| 4. | 7.11 | 320.0917[M]^+^ | C_19_H_14_NO_4_ | Coptisine |
| 5. | 7.13 | 336.1230[M]^+^ | C_20_H_18_NO_4_ | Epiberberine |
| 6. | 7.23 | 338.1392[M]^+^ | C_20_H_20_NO_4_ | Jatrorrhizine |
| 7. | 7.69 | 447.0922[M+H]^+^ | C_21_H_18_O_11_ | Baicalin |
| 8. | 8.25 | 352.1543[M]^+^ | C_21_H_22_NO_4_ | Palmatine |
| 9 | 8.29 | 336.1230[M]^+^ | C_20_H_18_NO_4_ | Berberine |
| 10 | 9.21 | 461.1078[M+H]^+^ | C_22_H_20_O_11_ | Wogonoside |
| 11 | 10.51 | 271.0601[M+H]^+^ | C_15_H_10_O_5_ | Baicalein |
| 12 | 12.35 | 285.0757[M+H]^+^ | C_16_H_12_O_5_ | Wogonin |
